# Increased carotid intima-media thickness and cardiometabolic risk factors are associated with *IL-6* gene polymorphisms in Mexican individuals: The Genetics of Atherosclerotic Disease Mexican study

**DOI:** 10.17305/bb.2023.9495

**Published:** 2024-04-01

**Authors:** Rosalinda Posadas-Sánchez, Ángel Rene López-Uribe, Juan Reyes-Barrera, Julian Ramírez-Bello, María del Rocio Martínez-Alvarado, Gilberto Vargas-Alarcón

**Affiliations:** 1 Department of Endocrinology, Instituto Nacional de Cardiología Ignacio Chávez, Mexico City, Mexico; 2 Department of Molecular Biology and Research Direction, Instituto Nacional de Cardiología Ignacio Chávez, Mexico City, Mexico

**Keywords:** Carotid intima-media thickness (CIMT), cardiometabolic risk factors, interleukin 6 (IL-6), polymorphisms, coronary artery disease (CAD)

## Abstract

Interleukin 6 (IL-6) is a cytokine implicated in the development of atherosclerosis. This study aimed to determine the association of three *IL-6* gene polymorphisms with increased carotid intima-media thickness (CIMT) and cardiometabolic risk factors. Three *IL-6* polymorphisms (rs1800795, rs2069827, and rs1800796) were analyzed in 178 individuals with increased CIMT (CIMT ≥ 75th percentile) and 906 individuals without increased CIMT (CIMT < 75th percentile). Logistic regression, adjusted for confounding variables, was employed to assess the associations. The rs1800796 polymorphism was significantly associated with an elevated risk of increased CIMT (OR ═ 1.354, *P*_additive_ ═ 0.016; OR ═ 1.803, *P*_recessive_ ═ 0.014; OR ═ 1.989, *P*_codominant2_ ═ 0.008). One haplotype (GCG) correlated with a higher risk of increased CIMT (OR ═ 1.288; *P* ═ 0.008), while another (GGG) demonstrated a reduced risk (OR ═ 0.773; *P* ═ 0.006). In individuals without increased CIMT, the rs2069827 polymorphism was associated with low risks of central obesity, hypoalphalipoproteinemia, and a low risk of presenting with high levels of total cholesterol (TC), non-high-density lipoprotein cholesterol (non-HDL-C), low-density lipoprotein cholesterol (LDL-C)/HDL-C index, apolipoprotein B, and gamma-glutamyl transpeptidase. The rs1800796 polymorphism was associated with a low risk of adipose tissue insulin resistance, and the rs1800795 was associated with a minimal risk of central obesity and hypoalphalipoproteinemia. Among those with increased CIMT, rs2069827 was associated with low risks of central obesity, hypertriglyceridemia, metabolic syndrome, and a high triglyceride (TG)/HDL-C index, while rs1800796 was associated with a low risk of fatty liver. Similar IL-6 concentrations were observed in both individuals with and without increased CIMT. In conclusion, the rs1800796 polymorphism is associated with increased CIMT, while the rs2069827 and rs1800795 are linked to cardiovascular risk factors.

## Introduction

Coronary artery disease (CAD) is a chronic, progressive, and multifactorial pathology with an important impact on global health. One of the primary consequences of atherosclerosis is cardiovascular disease (CVD). Traditional risk factors for CAD, such as obesity, hypertension, high total cholesterol (TC), type 2 diabetes mellitus, and smoking, have been extensively studied. However, these factors have proven to be weak predictors of CAD [1]. Currently, coronary angiography is regarded as the actual gold standard for evaluating coronary atherosclerosis. However, it is an invasive method with associated risks. Some studies have suggested that the carotid intima-media thickness (CIMT) serves as a rapid and reproducible marker for CAD [2, 3]. Tarnoki et al. [4] demonstrated the genetic influence on CIMT in a longitudinal twin study. Associations between increased CIMT and inflammation have been reported [5, 6]. Leonsson et al. [7] reported elevated interleukin 6 (IL-6) concentrations in patients with untreated pituitary and growth hormone deficiencies, and these concentrations positively correlated with CIMT. Similarly, IL-6 concentrations were linked to increased CIMT values in rheumatoid arthritis patients who did not have cardiovascular risk factors [8]. CIMT was associated with the *IL-6-174*, rs1800795 polymorphism in a prospective cohort study [9]. In this study, stroke-free individuals carrying the IL-6 GG genotype displayed an 11% higher increase in CIMT compared to individuals with the CC+GC genotypes. To date, no studies have examined the association between IL-6 levels, its polymorphisms, and the increase in CIMT among healthy individuals without a personal or family history of CAD. The objective of this study was to determine the association of these variables in a group of healthy Mexican individuals.

## Materials and methods

### Subjects

**Figure 1.**
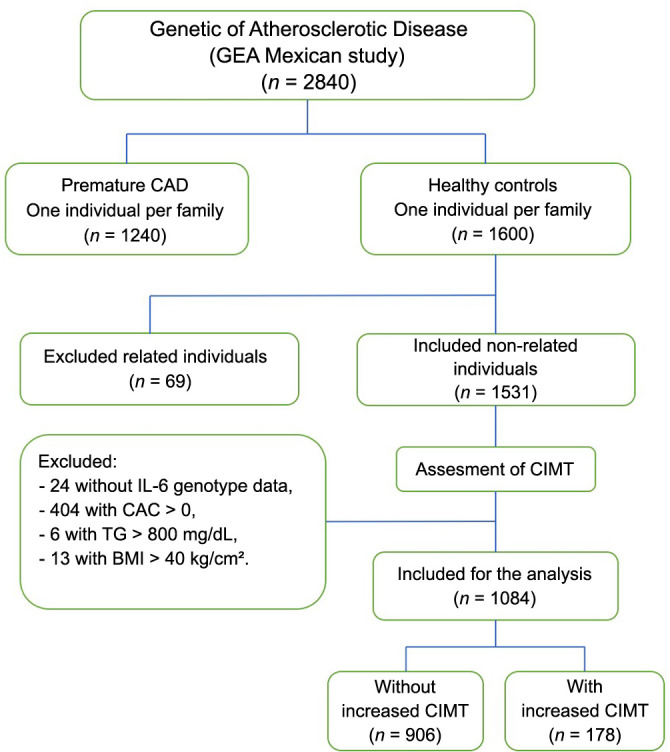
**Flowchart representing the disposition of the participants included in the GEA Mexican study.** The present study specifically incorporated the healthy participants who were not excluded based on the established exclusion criteria (178 with an increased CIMT and 906 without an increased CIMT). GEA: Genetics of Atherosclerotic Disease; CAD: Coronary artery disease; IL-6: Interleukin 6; CAC: Coronary artery calcification; TG: Triglycerides; BMI: Body mass index; CIMT: Carotid intima-media thickness.

The study included 178 individuals with an increased CIMT (IMT ≥ 75th percentile) and 906 without an increased CIMT (IMT < 75th percentile). These individuals were part of the control group of the Genetics of Atherosclerotic Disease (GEA) Mexican study (Figure 1). This group consisted of healthy individuals with no family history of premature CAD, and they were recruited at our Institute. All these participants underwent computed tomography (CT) of the chest and abdomen. The total, subcutaneous, and visceral abdominal fat were also determined in each participant, as reported by Kvist et al. [10]. Conversely, the coronary artery calcification (CAC) score was defined using the Agatston method [11]. The current study included only those individuals without CAC (CAC ═ 0). Characteristics of the individuals were determined as previously described [12–14].

### Measurement of the carotid intima-media thickness (CIMT)

The CIMT was measured using high-resolution ultrasound equipment in B mode (Sonosite Micromax) equipped with a 13–6 MHz linear transducer. The thickness of the intima and media layers was determined in a supine position with an extended neck. The measurements took into account the distance between the arterial intima-lumen and the media-adventitia interfaces of the far wall. CIMT was defined based on the average of five readings taken from both the left and right carotid arteries. A CIMT value above the 75th percentile was established in line with the standards set by the American Society of Echocardiography. These measurements were then adjusted for age and sex-specific percentiles tailored for ethnicity, specifically for this study, benchmarks were set according to the Hispanic population [15, 16]. A value above the 75th percentile was considered an increased CIMT.

### IL-6 concentrations

Serum IL-6 concentrations were determined using a Bioplex system (R&D Systems, Minneapolis, MN, USA), in accordance with the manufacturer’s instructions. Data analysis was conducted through the Bio-Plex Manager, and the results are presented in pg/mL.

### Genetics

Genomic DNA was obtained from whole peripheral blood employing the QIAamp DNA Blood Mini Kit (QIAGEN, Hilden, Germany). IL-6 genotypes rs2069827G/T, rs1800795G/C, and rs1800796G/C were identified using TaqMan assays on the 7900HT real-time PCR model (Applied Biosystems, Foster City, CA, USA). To ensure accuracy, 10% of the samples were assayed in duplicate, and the outcomes were consistent with the initial findings. The polymorphisms were selected based on a previous bioinformatics analysis we conducted using the SNPinfo program (https://snpinfo.niehs.nih.gov/snpinfo/snpfunc.html).

### Ethical statement

This research was conducted in accordance with the Declaration of Helsinki, and approved by the Ethics Committee of Instituto Nacional de Cardiología Ignacio Chávez (protocol 19-1104, 02-18-2019). All sample collections underwent the necessary approval from the Institutional Review Board.

### Statistical analysis

The data are presented as frequencies, medians (with interquartile range [IQR]), or means ± standard deviations. Categorical variables were compared using the chi-square test, while continuous variables were analyzed using the Student’s *t*-test or Mann–Whitney *U* test. The Hardy–Weinberg equilibrium was evaluated using the chi-square test. The associations between the *IL-6* polymorphisms and increased CIMT, as well as with cardiovascular risk factors, were determined using logistic regression analysis under various inheritance models (additive, codominant 1, codominant 2, dominant, heterozygous, and recessive). The models were adjusted for potential confounding variables as appropriate. Haplotype analysis and linkage disequilibrium evaluations were conducted using the Haploview version 4.1 program (https://www.broadinstitute.org/haploview/haploview) (Broad Institute of Massachusetts Institute of Technology and Harvard University, Cambridge, MA, USA). IL-6 concentrations in individuals with and without increased CIMT were evaluated using the Mann–Whitney or Kruskal–Wallis *U* test, as appropriate.

## Results

### Demographic, clinical, and biochemical characteristics

**Figure 2.**
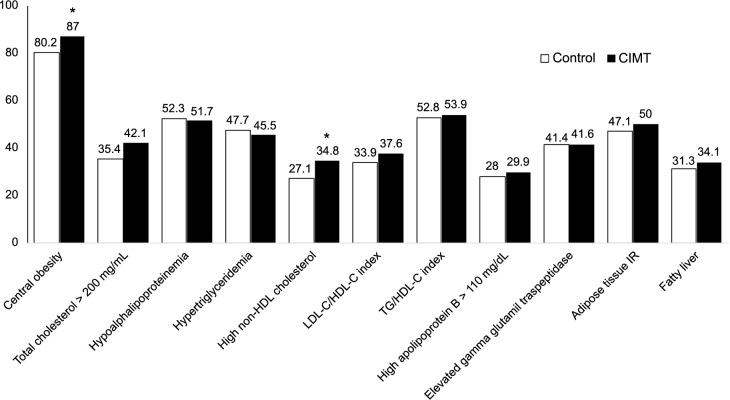
**Distribution of cardiovascular risk factors in individuals with increased CIMT (CIMT - black bars) and those without increased CIMT (control - white bars).** High prevalence of central obesity and high non-HDL-C was observed in individuals with increased CIMT**.** *P < 0.05. CIMT: Carotid intima-media thickness; non-HDL: Non-high-density lipoprotein; LDL-C: Low-density lipoprotein cholesterol; HDL-C: High-density lipoprotein cholesterol; TG: Triglycerides; IR: Insulin resistance.

According to their CIMT values, the individuals were categorized into two groups: those with CIMT ≥ 75th percentile (individuals with increased CIMT) and those with CIMT < 75th percentile (individuals without increased CIMT). Individuals with increased CIMT exhibited a higher body mass index (BMI) (*P* ═ 0.010), and higher concentrations of TC (*P* ═ 0.001), low-density lipoprotein (LDL) (*P* ═ 0.001), and non-high-density lipoprotein (non-HDL) (*P* ═ 0.004) compared to those without increased CIMT (Table 1). A high prevalence of central obesity and high non-HDL cholesterol (non-HDL-C) levels was observed in individuals with increased CIMT compared to those without increased CIMT (*P* < 0.05) (Figure 2).

**Table 1:** Demographic, clinical, and biochemical characteristics of the studied groups

	**Without increased CIMT (*n* ═ 906)**	**With increased CIMT** **(*n* ═ 178)**	P*
Age (years)	51 ± 9	53 ± 8	<0.001
Sex (% of men)	42.9	31.5	0.005
Body mass index (kg/m^2^)	27.8 (25.3 – 30.6)	28.3 (26.2 – 31.8)	0.010
Waist circumference (cm)	93.5 ± 11.2	95.0 ± 10.4	0.071
*Cholesterol (mg/dL)*			
Total	188 (165 – 209)	194 (176 – 224)	0.001
LDL-C	114 (94 – 133)	121 (103 – 141)	0.001
HDL-C	45 (36 – 54)	46 (37 – 57)	0.167
Non-HDL	141 (120 – 163)	147 (127 – 176)	0.004
Triglycerides (mg/dL)	146 (107 – 201)	141 (111 – 207)	0.620
LDL-C/HDL-C index	2.6 (2.0 – 3.3)	2.7 (2.0 – 3.4)	0.250
Triglycerides/HDL-C index	3.2 (2.1 – 5.3)	3.3 (2.1 – 5.0)	0.897
Apolipoprotein B (mg/dL)	93 (76 – 113)	95 (77 – 115)	0.524
Gamma gutamyl transpeptidase (U/L)	26 (18 – 44)	25 (18 – 40)	0.704
Adipose tissue insulin resistance	9.2 (5.9 – 13.9)	10.1 (6.8 – 15.9)	0.063
IL-6 (pg/mL)	0.87 (0.41 – 1.17)	0.79 (0.41 – 1.73)	0.422

Data are presented as mean ± standard deviation, median (interquartile range) or percentage. IL-6 concentrations were determined in 876 individuals without increased CIMT and in 174 with increased CIMT. *Student’s *t*-test, Mann–Whitney *U* test, or chi-squared test. CIMT: Carotid intima-media thickness; IL-6: Interleukin 6; LDL-C: Low-density lipoprotein cholesterol; HDL-C: High-density lipoprotein cholesterol; non-HDL: Non-high-density lipoprotein.

### IL-6 concentration and its association with IL-6 genotypes

IL-6 concentrations were similar in individuals with increased CIMT (0.79 pg/mL [IQR 0.41–1.73]) and those without increased CIMT (0.87 pg/mL [IQR 0.41–1.73]) (*P* ═ 0.422). Across the entire population, no association was found between the IL-6 concentrations and the IL-6 genotypes (*P* > 0.05) (Table S1).

### Association of *IL-6* polymorphisms with increased carotid intima-media thickness

**Figure 3.**
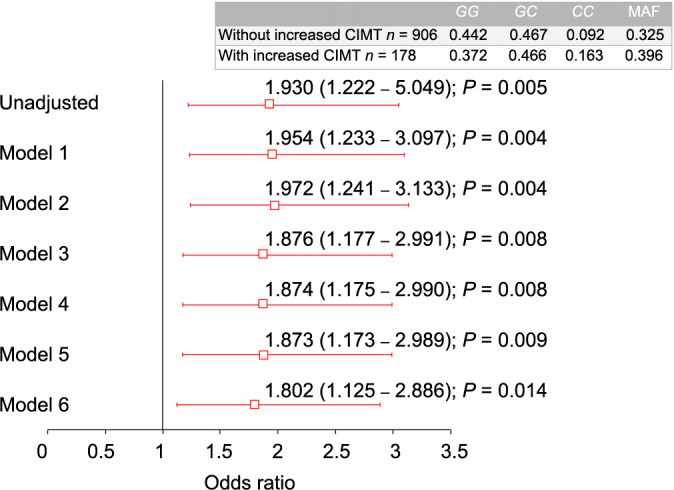
**Association of increased CIMT with *IL-6* rs1800796 polymorphism, under six models adjusted for confounding variables.** Model 1 is adjusted for age. Model 2 is adjusted for model 1 and sex. Model 3 is adjusted for model 2 and BMI. Model 4 is adjusted for model 3 and LDL-C levels. Model 5 is adjusted for model 4 and current smoking. Model 6 is adjusted for model 5 and type 2 diabetes mellitus. The data represent OR (95% CI). The *P* values were calculated using the logistic regression analysis. CIMT: Carotid intima-media thickness; IL-6: Interleukin 6; LDL-C: Low-density lipoprotein cholesterol; MAF: Minor allele frequency; BMI: Body mass index.

The three studied polymorphisms (rs2069827, rs1800795, and rs1800796) were in Hardy–Weinberg equilibrium (*P* > 0.05). A similar distribution of the rs2069827 genotype and the rs1800795 genotype was observed in individuals with and without increased CIMT (*P* > 0.05). However, the rs1800796 polymorphism was associated with a higher risk of increased CIMT under six models adjusted for confounding variables. Specifically, under the recessive model adjusted by age, sex, BMI, LDL-cholesterol (LDL-C), current smoking, and type 2 diabetes mellitus, individuals had a 1.8-fold higher risk of increased CIMT (OR ═ 1.802, 95% CI 1.125–2.886; *P* ═ 0.014) (Figure 3).

### Association of *IL-6* polymorphisms with cardiovascular risk factors in individuals without increased carotid intima-media thickness

**Figure 4.**
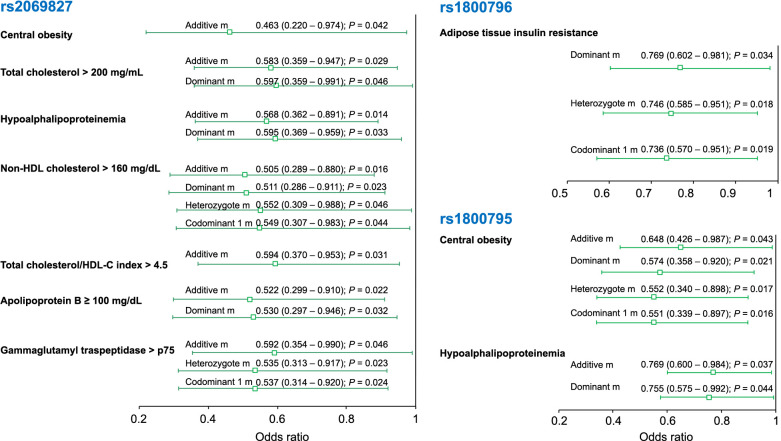
**Association of *IL-6* polymorphisms with cardiovascular risk factors in individuals without increased CIMT.** The association between *IL-6* polymorphisms and various variables under different inheritance models is presented. The data represent OR (95% CI). IL-6: Interleukin 6; CIMT: Carotid intima-media thickness; HDL: High-density lipoprotein.

In the group of individuals without increased CIMT, the rs2069827 polymorphism was associated with a decreased risk of central obesity (OR ═ 0.463, 95% CI 0.220–0.974; *P*_additive_ ═ 0.042), TC > 200 mg/mL (OR ═ 0.583, 95% CI 0.359–0.947; *P*_additive_ ═ 0.029; OR ═ 0.597, 95% CI 0.359–0.991; *P*_dominant_ ═ 0.046), hypoalphalipoproteinemia (OR ═ 0.568, 95% CI 0.362–0.891; *P*_additive_ ═ 0.014; OR ═ 0.595, 95% CI 0.369–0.959; *P*_dominant_ ═ 0.033), non-HDL-C > 160 mg/dL (OR ═ 0.505, 95% CI 0.289–0.880; *P*_additive_ ═ 0.016; OR ═ 0.511, 95% CI 0.286–0.911; *P*_dominant_ ═ 0.023; OR ═ 0.552, 95% CI 0.309–0.988; *P*_heterozygote_ ═ 0.046; OR ═ 0.549, 95% CI 0.307–0.983; *P*_codominant1_ ═ 0.044), LDL-C/HDL-C index > 4.5 (OR ═ 0.594, 95% CI 0.370–0.953; *P*_additive_ ═ 0.031), apolipoprotein B ≥ 100 mg/dL (OR ═ 0.522, 95% CI 0.299–0.910; *P*_additive_ ═ 0.022; OR ═ 0.530, 95% CI 0.297–0.946; *P*_dominant_ ═ 0.032), gamma-glutamyl-transpeptidase > 75th percentile (OR ═ 0.592, 95% CI 0.354–0.990; *P*_additive_ ═ 0.046; OR ═ 0.535, 95% CI 0.313–0.917; *P*_heterozygote_ ═ 0.023; OR ═ 0.537, 95% CI 0.314–0.920; *P*_codominant1_ ═ 0.024). On the other hand, the rs1800796 polymorphism was associated with a decreased risk of adipose tissue insulin resistance (OR ═ 0.769, 95% CI 0.602–0.981; *P*_dominant_ ═ 0.034; OR ═ 0.746, 95% CI 0.585–0.951; *P*_heterozygote_ ═ 0.018; OR ═ 0.736, 95% CI 0.570–0.951; *P*_codominant1_ ═ 0.019). Additionally, the rs1800795 polymorphism was associated with a decreased risk of central obesity (OR ═ 0.648, 95% CI 0.426–0.987; *P*_additive_ ═ 0.043; OR ═ 0.574, 95% CI 0.358–0.920; *P*_dominant_ ═ 0.021; OR ═ 0.552, 95% CI 0.340–0.898; *P*_heterozygote_ ═ 0.017; OR ═ 0.551, 95% CI 0.339–0.897; *P*_codominant1_ ═ 0.016), and hypoalphalipoproteinemia (OR ═ 0.769, 95% CI 0.600–0.984; *P*_additive_ ═ 0.037; OR ═ 0.755, 95% CI 0.575–0.992; *P*_dominant_ ═ 0.044) (Figure 4).

### Association of IL-6 polymorphisms with cardiovascular risk factors in individuals with increased carotid intima-media thickness

**Figure 5.**
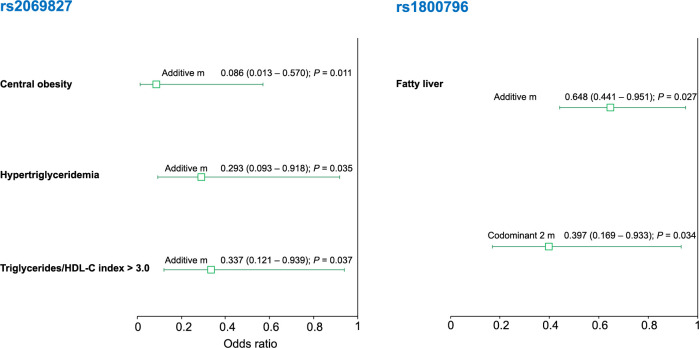
**Association of *IL-6* polymorphisms with cardiovascular risk factors in individuals with increased CIMT.** The association between *IL-6* polymorphisms and various variables under different inheritance models is presented. The data represent OR (95% CI). IL-6: Interleukin 6; CIMT: Carotid intima-media thickness; HDL-C: High-density lipoprotein cholesterol.

In the group of individuals with increased CIMT, under the additive model, the rs2069827 polymorphism was associated with a decreased risk of central obesity (OR ═ 0.086, 95% CI 0.013–0.570; *P* ═ 0.011), hypertriglyceridemia (OR ═ 0.293, 95% CI 0.093–0.918; *P* ═ 0.035), and triglycerides (TG)/HDL-C index > 3.0 (OR ═ 0.337, 95% CI 0.121–0.939; *P* ═ 0.037). On the other hand, the rs1800796 polymorphism was associated with a decreased risk of fatty liver (OR ═ 0.648, 95% CI 0.441–0.951; *P*_additive_ ═ 0.027; OR ═ 0.397, 95% CI 0.169–0.933; *P*_codominant2_ ═ 0.034) (Figure 5).

## Discussion

In the present study, analyzing the *IL-6* polymorphisms in individuals with and without increased CIMT, we found that the rs1800796 C allele and the GCG haplotype were associated with an increased risk of elevated CIMT, whereas rs2069827 and rs1800795 were associated with certain cardiovascular risk factors.

Patients with CAD exhibit elevated levels of inflammatory markers, including specific cytokines such as IL-6 [17, 18]. IL-6 plays an important role as a central regulator of the inflammatory process, acting as an inducer of acute phase response [19]. It has been reported that IL-6 is a crucial factor in the early onset of atherosclerosis and has been proposed as a marker indicating a broader extent of atherosclerotic lesions [20]. However, the correlations between IL-6 concentrations and CIMT are controversial, with studies yielding both positive and negative findings. For instance, a direct relationship between IL-6 concentrations and CIMT in patients with type 2 diabetes mellitus has been reported [21]. Conversely, Elkind et al. [22], when examining stroke-free individuals, observed correlations with IL-2 concentrations but not with IL-6 levels in relation to IMT. In individuals with subclinical atherosclerosis, a positive correlation between IL-6 levels and CIMT was identified [23]. Furthermore, a meta-analysis comprising 37 studies found that the IL-6 levels significantly correlated with IMT in patients with CVD, those at risk of CVD, and even healthy participants [24]. Notably, in this meta-analysis, only two studies focused on healthy participants [25, 26]. While all these studies identified a correlation between IL-6 levels and CIMT, none of them analyzed the relationship between the two variables. In our study, in healthy individuals (without subclinical atherosclerosis as assessed by CT), IL-6 concentrations did not differ significantly between subjects with increased CIMT and those without. To the best of our knowledge, this study is the first to evaluate the relationship between IL-6 concentrations and CIMT in healthy individuals.

The CIMT serves as a noninvasive, rapid, and reproducible marker for CAD. Evidence has underscored the genetic influence on both CIMT and arterial stiffness. Investigating polymorphisms in genes linked to elevated CIMT is essential for identifying potential genetic markers for this condition. Several genetic polymorphisms have been associated with CAD, yet it remains unclear whether these same polymorphisms are also linked to carotid atherosclerosis. Cunnington et al. [27] reported that six polymorphisms (rs1333049, rs7044859, rs496892, rs7865618, rs6922269, and rs2943634) previously associated with CAD did not correlate with CIMT. Similarly, two polymorphisms found in the toll-like receptor 4 (*TLR-4*) gene showed no association with carotid atherosclerosis in the general population [28]. In research conducted by our team, we found no linkage between *IL-6* polymorphisms and the risk of premature CAD (submitted for publication). However, in the current study focused on healthy individuals, the rs1800796 C allele and the GCG (rs2069827, rs1800796, and rs1800795*)* haplotype were found to be associated with an increased risk of elevated CIMT. Notably, the risk haplotype is defined by the presence of the rs1800796 C risk allele. Hence, identifying this particular polymorphism is sufficient to discern the association. Rundek et al. [9], when studying stroke-free participants, reported that those possessing the IL-6 GG (rs1800795) genotype had a CIMT that was 11% greater than participants with other genotypes. Similarly, Ou et al. [29], in their study of young patients with internal carotid stenosis, found that IL-6 promoter polymorphisms heightened the risk of recurrent stroke. Another study identified a connection between the IL-6 GG (rs1800795) genotype and increased CIMT in asymptomatic individuals with carotid atherosclerosis [30]. In our research, this polymorphism was not independently associated with elevated CIMT. Nonetheless, the risk haplotype does encompass the G allele of this polymorphism, aligning with findings by Rundek et al. [9] and Rauramaa et al. [30]. The rs1800796 is situated in the promoter region of the *IL-6* gene. Bioinformatics tools suggest that the C allele in this polymorphism modifies the binding affinity for transcription factors fetal alz-50 clone 1 (FAC1) and lymphoma/leukemia-related factor (LRF). This may lead to heightened IL-6 expression, explaining the association of this polymorphism with CIMT elevation. Additionally, this polymorphism has been linked to an increased CAD risk in both Asian and Caucasian populations [31].

In individuals without elevated CIMT, the rs2069827 T allele was associated to a reduced risk of central obesity, hypoalphalipoproteinemia. It was also associated with a low risk of presenting with high levels of TC, non-HDL-C, the LDL-C/HDL-C index, apolipoprotein B, and gamma-glutamyl transpeptidase. This polymorphism is situated in the promoter region of the *IL-6* gene. According to bioinformatics tools, the T allele modifies the binding affinity for the hepatocyte nuclear factor 3 beta (HNF3B) transcription factor, impacting the gene’s transcription and subsequent protein production. The rs1800796 C allele correlated with a decreased risk of adipose tissue insulin resistance. Meanwhile, the rs1800795 C allele was linked to a low risk of central obesity and hypoalphalipoproteinemia. Both of these polymorphisms are located in the promoter region and modify the binding affinity for specific transcription factors: the rs1800796 C allele for LRF and the rs1800795 C allele for the nuclear factor I (NFI), tumor protein p53 (tp53), and yin yang 1 (YY1). In individuals with elevated CIMT, the rs2069827 was associated with a low risk of central obesity, hypertriglyceridemia, metabolic syndrome, and a high TG/HDL-C index. The rs1800796 was associated with a low risk of fatty liver.

The distribution of the alleles for these polymorphisms varies according to the population being studied. This variation could influence the associations observed in the present research. Based on data from the National Center for Biotechnology Information, individuals of Mexican (from Los Angeles, CA, USA), Caucasian, African, and Asian heritage are less likely to possess the rs2069827 T allele, with frequencies of 2.3%, 11.6%, 0.3%, and 0.1%, respectively. In contrast, the frequencies for the G allele in these populations are 97.7%, 88.4%, 99.7%, and 99.9%, respectively. Similarly, Caucasians, Africans, Asians, and those of Mexican ancestry present a lower frequency of the rs1800795 C allele, with frequencies of 41.6%, 1.8%, 0.1%, and 13.3%, respectively, when compared to the G allele, which has frequencies of 58.4%, 98.2%, 99.9%, and 86.7%, respectively. The rs1800796 C allele is notably frequent in Asians (79%) but less so in Mexicans (34.4%), Caucasians (5%), and Africans (10%) (https://www.ensembl.org/index.html). Given this variance, we recommend studies involving populations with diverse genetic backgrounds to elucidate the true impact of these polymorphisms on the risk of increased CIMT and associated cardiovascular risk factors.

One of the key strengths of this study is its inclusion of healthy individuals without CAC, as measured by CT, and who have no personal and family history of CVD. The subjects are part of a well-structured Mexican cohort with detailed clinical, demographic, biochemical, and tomographic profiles. However, some limitations must be considered. Firstly, this is a non-prospective, cross-sectional study whose design does not allow for the establishment of causality. Secondly, the number of individuals with increased CIMT was limited, preventing stratification by sex. As such, caution should be exercised when interpreting the results. Another limitation is the absence of experiments that might elucidate the mechanisms by which these polymorphisms impact the disease process.

While the association of risk factors with the development of coronary and carotid artery disease is established, our study offers valuable insights into the association of *IL-6* polymorphisms with these risk factors in individuals both with and without increased CIMT. It is worth noting that the CIMT values deemed as normal can vary based on population, sex, and age. Therefore, any replication of the associations identified in this study should take these variations into account.

## Conclusion

In conclusion, our study suggests that the increase in CIMT may be influenced by the presence of the rs1800796 C allele, which could be viewed as a risk factor for this elevation. Both the rs2069827 and rs1800795 polymorphisms are associated with various cardiovascular risk factors in individuals with and without elevated CIMT. Consequently, the rs2069827, rs1800795, and rs1800796 polymorphisms could serve as potential genetic markers for an increase in CIMT and associated cardiovascular risk factors.

## Supplemental data

**Table S1:** IL-6 levels of the entire studied population, stratified by the *IL-6* gene polymorphism

**Polymorphism**	**Genotypes**			*P*
**rs2069827**	**GG**	**GC**	**CC**	
*n*	955	71	2	
IL-6 (pg/mL)	0.86 (0.40 – 1.73)	0.83 (0.50 – 1.81)	2.62 (0.29 – 4.95)	0.852
**rs1800796**	**GG**	**GC**	**CC**	
*n*	442	485	101	
IL-6 (pg/mL)	0.83 (0.41 – 1.77)	0.86 (0.41 – 1.72)	0.86 (0.40 – 1.63)	0.868
**rs1800795**	**GG**	**GT**	**TT**	
*n*	776	238	14	
IL-6 (pg/mL)	0.82 (0.40 – 1.71)	0.94 (0.44 – 1.86)	0.97 (0.78 – 1.29)	0.312

Data are presented as median (interquartile range). This analysis was made on 1028 individuals. IL-6: Interleukin 6.

## Data Availability

The data analyzed in this study are not publicly accessible due to institutional policy; however, they are available upon reasonable request from the corresponding author.
